# Graded Response Model Analysis and Computer Adaptive Test Simulation of the Depression Anxiety Stress Scale 21: Evaluation and Validation Study

**DOI:** 10.2196/45334

**Published:** 2023-06-22

**Authors:** Jake Kraska, Karen Bell, Shane Costello

**Affiliations:** 1 School of Educational Psychology and Counselling Faculty of Education Monash University Clayton Australia

**Keywords:** graded response model, DASS-21, CAT, computer adaptive testing, simulation, psychological distress, depression, anxiety, stress, simulation, mental health, screening tool, tool, reliability, development, model

## Abstract

**Background:**

The Depression Anxiety Stress Scale 21 (DASS-21) is a mental health screening tool with conflicting studies regarding its factor structure. No studies have yet attempted to develop a computer adaptive test (CAT) version of it.

**Objective:**

This study calibrated items for, and simulated, a DASS-21 CAT using a nonclinical sample.

**Methods:**

An evaluation sample (n=580) was used to evaluate the DASS-21 scales via confirmatory factor analysis, Mokken analysis, and graded response modeling. A CAT was simulated with a validation sample (n=248) and a simulated sample (n=10,000) to confirm the generalizability of the model developed.

**Results:**

A bifactor model, also known as the “quadripartite” model (1 general factor with 3 specific factors) in the context of the DASS-21, displayed good fit. All scales displayed acceptable fit with the graded response model. Simulation of 3 unidimensional (depression, anxiety, and stress) CATs resulted in an average 17% to 48% reduction in items administered when a reliability of 0.80 was acceptable.

**Conclusions:**

This study clarifies previous conflicting findings regarding the DASS-21 factor structure and suggests that the quadripartite model for the DASS-21 items fits best. Item response theory modeling suggests that the items measure their respective constructs best between 0θ and 3θ (mild to moderate severity).

## Introduction

The Depression Anxiety Stress Scale (DASS) [1] measures symptoms of depression, anxiety, and stress. The DASS-21 has robust psychometric properties, and computer adaptive test (CAT) and item response theory (IRT) pose an opportunity to further improve the DASS-21 and better understand “psychological distress.”

### The DASS-21

Since its inception, the DASS has been refined from 42 items (DASS-42) to 21 items (DASS-21). The DASS-42 contained 3 scales of 14 items each, while the DASS-21 contains 3 scales of 7 items each. The reduction from 14-item to 7-item scales was possible while maintaining adequate psychometrics [2]: reduction from .97, .92, and .95 to .94, .87, and .91 for the depression, anxiety, and stress scales, respectively. Support for the 3-factor structure has been found in both clinical [3] and nonclinical samples [4]. Some have suggested the DASS-21 measures an additional “general psychological distress” factor [5,6], while others have suggested it is “more adequately explained by a 2-factor model of physiological arousal and generalized negativity” [7].

As has been noted by other researchers, there have been inconsistences relating to terminology regarding the factor structure of the DASS [8]. In the context of the DASS, a “bifactor” model has sometimes been referred to as a “quadripartite” model as there is 1 general factor (overall negative affect factor) with 3 specific factors (stress, anxiety, and depression). A bifactor model is a latent structure, where a general factor loads on all items and other factors load on different sets of items and explain unique response variance that is not accounted for by the general factor [9]. This differs from a hierarchical model, whereby the general factor sits “above” the specific factors.

For the DASS-21, Henry and Crawford [5] found that a quadripartite model demonstrated better fit than one that removed the stress factor and allowed the stress items to freely load on a negative affect general factor (the “tripartite model”). The quadripartite model has been supported when using the DASS-21 with adolescents [10] and adults [11]. This structure seems to overcome many of the disagreements about an underlying negative affect construct within the DASS-21. Outside of factor structure and reliability, differences in item response patterns based on demographics (ie, differential item functioning [DIF]) have not been found for the DASS-21 across age, gender, education, mode of administration, or disability [11-14].

### Item Response Theory

The DASS-21 was developed from the perspective of classical test theory (CTT). There is an assumption that each observed score (*X*) is made up of the true score (*T*) and random error (*E*). CTT relies heavily on the concept of internal consistency; items with low total score correlations and items that are endorsed infrequently are removed, increasing the variance of the total score relative to the number of items, which increases the reliability [15]. However, all items must be administered to obtain a score, and as items at each end of the spectrum are eliminated, the scale discriminates poorly among those with extreme trait levels.

In contrast, IRT allows the modeling of item response probabilities. This allows the determination of reliability (“information” in IRT) and trait level (θ) of each item allowing score calculations for different item sets [16]. Using different IRT models, item characteristics can be plotted, and item sets that measure specific trait levels can be developed [17,18]. Understanding item characteristics allows researchers to ensure efficiency by using a smaller set of items and testing a wider range of a trait.

Some authors have analyzed the DASS-21 and DASS-42 using different IRT models. Parkitny and colleagues [13] were able to fit each of the scales to the Rasch model, but an aggregate measure of “negative affect” lacked fit. Medvedev and colleagues [12] used the Partial Credit Rasch model; several item calibrations (removal of item 5 in the depression scale, combining items 2 and 20, and 9 and 19, from the anxiety scale into “super items”) were carried out to improve the fit to the model. Shea and colleagues [14] also used the Partial Credit Rasch Model but needed to remove an item from each scale to reach an acceptable fit. Shea and colleagues [14] also suggested that the items could be used to form a revised 2-factor model, in line with Duffy and colleagues [7]. Wardenaar and colleagues [19] used the graded response model with a large sample of Dutch adults who had completed the DASS-42; they found that each scale was sufficiently unidimensional and that an unconstrained graded response model fits the data; however, items only measured in the mild to moderate range of severity. The studies conducted suggest that there is some use in using IRT with the DASS-21 items to further understand the location of items on the latent trait and how well they discriminate among individuals; this is particularly useful when attempting to understand severity of symptoms.

### CAT

One key advantage of IRT is that once a calibrated bank of items has been established, it is possible to use these items in a CAT. A CAT allows “item responses to be scored immediately and adaption to occur after each item is administered” [15]. That is, a person’s trait level can be re-estimated after each response. With a bank of items and implementation of various start rules, stop rules, estimation methods, and item selection procedures, it is possible to create fully automatable and highly efficient tests [15]. When starting a CAT, the first item can be selected based on what is known about the respondent or about the most informative item; alternatively, start items can be chosen at random to reduce the chances of respondents remembering test items. Once the CAT has started, various methods can estimate the respondent’s θ after each response [20]. The next item selected is the one that provides the most information based on the respondent’s prior response [21]. Lastly, a stop rule can be selected; these can include SE termination, minimum information termination, SE reduction criterion, and a change in θ criterion [22]. By using start rules, stop rules, estimation methods, and item selection procedures, almost any test can be turned into a CAT.

### Aims and Hypotheses

To our knowledge, the graded response model and CAT have not yet been used with the DASS-21. Moving from IRT of short mental health scales to CAT development has been exampled using the Center for Epidemiological Studies Depression scale (CES-D) [23], General Anxiety Disorder-7 (GAD-7), Patient Health Questionnaire-9 (PHQ-9) and Perceived Stress Questionnaire (PSQ) [24], and the Beck Depression Inventory (BDI) [25]. Frequently, clinicians and researchers use a range of questionnaires in their work, increasing the time required from examinees. The integration of tools via CAT poses opportunities for efficiencies. This study aimed to determine whether the DASS-21 fits the graded response model and then validate its use as a CAT. An Australian nonclinical sample completed the DASS-21 and 2 simulations of the CAT using simulated responses, and real data were completed. It is expected that simulated CATs will be able to be administered with fewer items while maintaining acceptable levels of reliability.

## Methods

### Ethics Approval

An ethics exemption for use of prior data was provided by the Monash University Human Research Ethics Committee in May 2018 (project 13909). All procedures for data collection were consistent with the Helsinki Declaration of 1975, as revised in 2000. The procedure for data collection for the original research is included below.

### Participants

Participants (n=828) between the ages of 18 and 84 years were recruited as part of a larger study that included questionnaires on dark triad traits, mental health, and cognitive style. Questionnaires were administered via Qualtrics. Participants were recruited via Facebook advertising and social media snowball sampling. Participants could provide their email addresses and enter a draw to win 1 of 3 Westfield Aus $50 (US $37) vouchers. There were no missing data.

The sample was randomly divided into 2 groups. The first sample was used for evaluation of the DASS-21, item calibration, and deriving the item parameters (n=580). The second was used for validating the developed models and for the CAT simulation (n=248). A simulated sample (n=10,000) was also used for the CAT simulation. Table 1 summarizes the demographics of the total sample. The mean age was 51 (SD 14.25) years. The proportion of group membership was maintained across the split. There were no significant differences between the evaluation and validation sample using a Welch 2-sample *t* test for depression (*t*_429_=−1.31; *P*=.19; 95% CI −1.17 to 0.23; anxiety, *t*_403_=−0.98; *P*=.33; 95% CI −0.82 to 0.27; and stress, *t*_407_=−1.64; *P*=.10; 95% CI −1.23 to 0.11).

**Table 1:** Participant demographics.^a^

Characteristic			Total sample, n (%)		Evaluation sample, n (%)		Validation sample, n (%)		
**Gender**									
	Female	662 (79.95)		467 (80.52)		195 (78.63)			
	Male	166 (20.05)		113 (19.48)		53 (21.37)			
**Age group (years)**									
	18-29	96 (11.59)		64 (11.03)		32 (12.90)			
	30-39	85 (10.27)		61 (10.52)		24 (9.68)			
	40-49	117 (14.13)		83 (14.31)		34 (13.71)			
	50-59	269 (32.49)		185 (31.90)		84 (33.87)			
	60-69	202 (24.40)		149 (25.69)		53 (21.37)			
	70-79	57 (6.88)		36 (6.21)		21 (8.47)			
	≥80	2 (0.24)		2 (0.34)		0 (0)			
**Employment**									
	Student	57 (6.88)		38 (6.55)		19 (7.66)			
	Not currently in workforce	65 (7.85)		46 (7.93)		19 (7.66)			
	Part-time or full-time work	541 (65.34)		382 (65.86)		159 (64.11)			
	Retired	165 (19.93)		114 (19.65)		51 (20.56)			
**Qualification**									
	Bachelor’s degree	230 (27.78)		166 (28.62)		64 (25.81)			
	Completed high school	107 (12.92)		70 (12.07)		37 (14.92)			
	Did not complete high school	49 (5.92)		36 (6.21)		13 (5.24)			
	Honors or graduate diploma	139 (16.79)		101 (17.41)		38 (15.32)			
	Masters or PhD	152 (18.36)		107 (18.45)		45 (18.14)			
	TAFE^b^ certificate or diploma or trade	151 (18.24)		100 (17.24)		51 (20.56)			

^a^There was no statistically significant difference between groups by age; *t*(483.7)=−1.41; *P*=0.16; 95% CI −3.59 to 0.59.

^b^TAFE: Technical and Further Education.

### Measure

The DASS-21 is a self-report questionnaire with 3 scales that measures depression, anxiety, and stress. Participants responded to the DASS-21 by indicating how much a statement applied to them over the past week on a 4-point Likert scale. Each scale has a potential range of 0 to 21, with higher scores indicating more severe symptomology. While labels can be used to characterize the degree of severity relative to the population, for research, it is preferable that the quantitative outcomes be used [26].

The DASS-21 was developed in Australia and is gaining popularity internationally [19]. The scale has good reliability, with Cronbach α coefficients for the total scale, and the scales ranging from .82 to .97 [1,2,27].

### Data Analysis

#### Software

Analyses [28] were conducted using R (version 4.0.2; R Foundation for Statistical Computing) [29] within RStudio (version 1.3.1073; Posit) [30].

#### Reliability

Reliability was measured using Cronbach α [31] for the total scale and each scale using the *psych* package for R (version 2.0.7) [32].

#### Confirmatory Factor Analysis

Confirmatory factor analysis (CFA) was completed to (1) clarify the factor structure described in the literature and (2) confirm that each scale was unidimensional. The chi-square statistic [33] has been found to be biased for large samples; thus, fit indices are also used, relying upon several cutoff criteria. The comparative fit index (CFI) [34] and the Tucker Lewis index (TLI) [35] measure whether the designated model fits the data better than a restricted model, with greater than 0.9 being considered an acceptable fit [36]. Some authors argue that the root-mean-square error of approximation (RMSEA) [37], which determines how closely the model replicates covariances, is more appropriate than the CFI for confirmatory analyses [38]; values below 0.01 are considered excellent, below 0.05 considered good, and below 0.08 considered mediocre [39]. The standardized root-mean-square residual (SRMR) [36] represents the average discrepancy between an implied correlation matrix and the observed correlation matrix; a value less than 0.08 is considered good [40]. The *lavaan* package for R (version 0.6-7) [41], relying on the diagonally weighted least squares estimator, was used.

#### Mokken Analysis

Mokken analysis [42] was carried out to determine if the items from each individual scale fit a Mokken scale; this assumes unidimensionality and an increasing “level” of the underlying trait (known as monotonicity). A Mokken scale indicates that a respondent is *more likely* to endorse a subsequent item based on a stronger agreement with prior items. Items that receive a Loevinger *H* value [43] of below 0.3 are considered to be inaccurate, between 0.3 and 0.4 considered to have low accuracy, between 0.4 and 0.5 have moderate accuracy, and values over 0.5 suggest “good” ordering [44,45]. Mokken scale analysis can be carried out if N>250, and the items are high quality [46]. Automatic item selection procedures also perform well with small sample sizes. Mokken analysis was carried out using the *mokken* package for R (version 3.0.2) [47,48].

#### Local Independence

Yen’s [49] Q3 method of correlated residuals was used to test the local independence of items. While item residual correlations above 0.20 are indicative of local dependence, some authors suggest that no singular critical value is appropriate for all situations [50]. Any items with local dependence would have their item information curves analyzed, and items with lower information would be removed. Local independence was analyzed using the *stats* package for R (version 4.0.2) [29].

#### Graded Response Model

The graded response model [51] was used, which is suitable for polytomous items [52]. The graded response model provides difficulty or trait-level parameters for *n* response categories (b*_n_*) and a discrimination parameter (a) for each item [53]. A response category corresponds to the number of response options on a scale; for the DASS-21, there are 4 response options for each item. The assumptions of unidimensionality, monotonicity, local independence, and item parameter invariance [54-56] were tested (as described earlier).

The hybrid C2 statistic [57] was used to test IRT model fit as there were no sufficient degrees of freedom to compute the M2* statistic. Along with the C2 statistic, associated fit indices such as TLI, CFI, RMSEA, and SRMR were computed. For item fit, the S-X^2^ [58] was used, and the RMSEA is reported to help evaluate the magnitude of item misfit.

Item discrimination parameters were interpreted in line with Baker and Kim [17]. Each item’s difficulty parameter is indicative of the point on the scale where the probability of endorsement is .5. Item thresholds were also analyzed. This is an important concept in psychological measurement that the probability of a response in one category is larger than that of any other single category [59], known as “ordered thresholds.” When there is a “discordance between the category probabilities and the underlying trait” [60], there are “disordered thresholds.” Where a disordered threshold occurs, a person with that level of θ may respond in an unexpected pattern. The *mirt* package for R (version 1.32.1) [61] was used for the IRT analyses.

#### Differential Item Functioning

Invariance in the item parameters was assessed by using DIF and differential test functioning. DIF occurs when the probability of endorsing an item or getting an item correct is different for different subgroups [62,63]. DIF was carried out using collapsed gender, employment, and education categories, using the *mirt* package for R (version 1.32.1) [61].

#### CAT Simulation

To determine the effectiveness of a CAT using the DASS-21 items, two simulations were carried out: (1) simulated responses (normal distribution of 10,000 participants with a mean θ of 0 and an SD of 1 were generated) and (2) a validation sample of 248 real participants. Correlations between the “true θ” and the CAT “simulated θ,” average number of items administered, and the amount of bias between simulated and real θ estimation were obtained.

Maximum posterior–weighted information was used for first item selection and subsequent item selection. For an item bank of this size, most item selection methods are considered appropriate, and maximum posterior–weighted information is much more straightforward [64]. The method for estimating θ after each item has been administered was expected a posteriori estimation, which produces less bias, particularly for smaller test lengths [65]. Simulations were carried out with a stop rule for 6 different levels of SE of measurement (SEM), corresponding with reliabilities of 1.0 (SEM~0.00), 0.9 (SEM~0.32), 0.8 (SEM~0.45), 0.7 (SEM~0.55), 0.6 (SEM~0.63), and 0.5 (SEM~0.71). The *mirtCAT* package for R (version 1.10) [66] was used for simulation.

## Results

### Reliability

The Cronbach α for the total DASS-21 using the evaluation sample was .94. Excellent internal consistency was found for the depression scale (.91), and good internal consistency was found for the anxiety (.80) and stress (.87) scales.

### Confirmatory Factor Analysis

In total, 9 different models were evaluated using the evaluation sample. Model A1 reflected a general psychological distress factor. Models B1 and B2 used the original 3-factor model. Models C1 and C2 combined the stress and anxiety traits [67]. Models D1 and D2 reflected the tripartite model [5]. Models E1 and E2 examined the fit of a quadripartite model also examined in Henry and Crawford [5]; unlike their study, E1 allowed shared variance between the factors.

**Figure 1:**
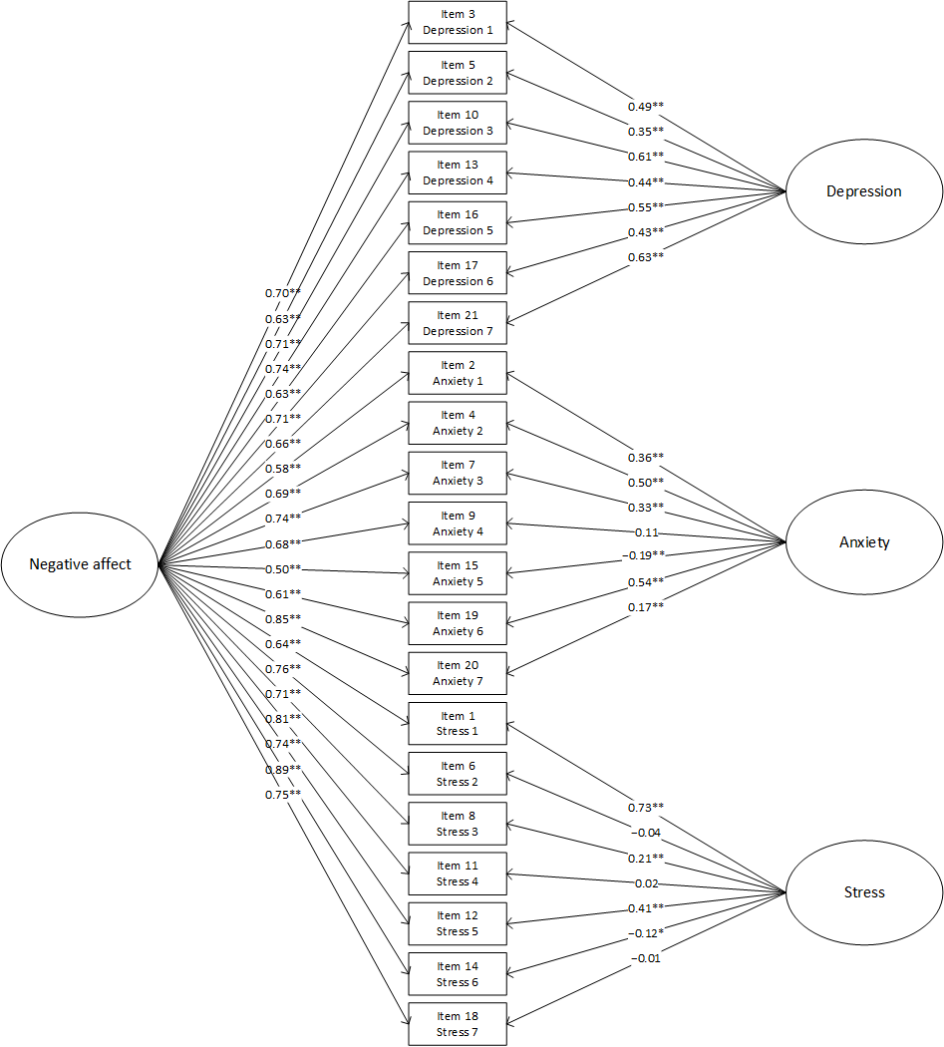
Bifactor model for the evaluation data. *P<.05, **P<.001.

Overall, the fit indices (n=580; Table 2) show that model E1 displayed the best fit; however, as this was not statistically significantly different from model E2 (Figure 1; *χ*^2^_3_=2.5; *P*=.47), E2 was retained as it is the simpler and more parsimonious model of the 2. Further, the same structure demonstrated good fit using the validation sample (n=248; Table 2). Item loadings were strong for the negative affect general factor, with only ANX4 (item 9; “I was worried about situations in which I might panic and make a fool of myself”), STR 2 (item 6; “I tended to overact to situations”), and STR 4 (item 11; “I found myself getting agitated”) demonstrating weaker loadings on the individual group factors.

Given the continued importance of 3 underlying traits and the unidimensionality requirement of IRT, each scale was analyzed independently (Table 3). All scales showed good to excellent fits, with only the RMSEA value for the stress scale being above the set cut off. The item factor loadings were generally strong, with all but 1 item above 0.7.

**Table 2:** Fit indices for the confirmatory factor analyses (n=580).^a^

Models		Fit index				Chi-square (*df*)	*P* value
		CFI^b^	TLI^c^	RMSEA^d^	SRMR^e^		
**Initial model analyses (model comparison)**							
	A1: One factor	0.98	0.98	0.09	0.09	1077.8 (189)	<.001
	B1: Three factors with shared variance	0.99	0.99	0.05	0.06	478.2 (186)	<.001
	B2: Three orthogonal factors	0.56	0.51	0.43	0.40	20,519.5 (189)	<.001
	C1: Two factors with shared variance	0.99	0.99	0.06	0.06	528.2 (188)	<.001
	C2: Two orthogonal factors	0.71	0.68	0.35	0.33	13,441.8 (189)	<.001
	D1: Tripartite model with shared variance	0.99	0.99	0.04	0.05	360.1 (174)	<.001
	D2: Tripartite model with orthogonal factors	0.99	0.99	0.04	0.05	371.9 (175)	<.001
	E1: Quadripartite model with shared variance	0.99	0.99	0.02	0.04	190.2 (165)	.08
	E2: Quadripartite model with orthogonal factors	0.99	0.99	0.02	0.04	193.9 (168)	.08
**Sample comparison^f^**							
	Model E2: Evaluation sample (n=580)	0.99	0.99	0.02	0.04	193.9 (168)	.08
	Model E2: Validation sample (n=248)	0.99	0.99	0.01	0.04	139.7 (168)	.94

^a^For each model pair, the first model allowed shared variance between depression, anxiety, and stress, whereas the second model forced these factors to be orthogonal; in the tripartite and quadripartite models, negative affect was always kept orthogonal.

^b^CFI: comparative fit index.

^c^TLI: Tucker Lewis index.

^d^RMSEA: root-mean-square error of approximation.

^e^SRMR: standardized root-mean-square residual.

^f^Using an orthogonal quadripartite model.

**Table 3:** Scale Confirmatory Factor Analysis and Graded Response Model analyses (n=580).

Fit index			Scale				
			Depression		Anxiety		Stress
**Confirmatory factor analysis**							
	Chi-square (*df*=14)	19.9		28.3		119.0	
	Comparative fit index (CFI)	0.99		0.99		0.98	
	Tucker Lewis index (TLI)	0.99		0.99		0.97	
	Root-mean-square error of approximation (RMSEA)	0.03		0.04		0.11	
	Standardized root-mean-square residual (SRMR)	0.03		0.05		0.07	
**Mokken analysis**							
	Scale Loevinger *H*	0.67		0.43		0.55	
	Scale Loevinger *H* standard error	0.02		0.03		0.02	
**Graded response model**							
	C2	42.16		46.47		157.87	
	CFI	0.99		0.98		0.95	
	TLI	0.99		0.97		0.93	
	RMSEA	0.06		0.06		0.13	
	SRMR	0.03		0.05		0.06	
	Marginal reliability	0.80		0.69		0.82	

### Mokken Analysis

Mokken analysis using the evaluation sample resulted in a Loevinger *H* of 0.511 (SE 0.024). The automatic item selection procedure did not suggest removal of any items. All items had a Loevinger *H* above 0.3, with a majority above 0.5.

For the individual scales, Mokken analysis revealed moderate accuracy for the anxiety scale and good ordering for the depression and stress scales (Table 3). The automatic item selection procedure did not suggest removal of any items. All items had a Loevinger *H* above 0.3, with a majority above 0.5.

### Local Independence

The results of Yen’s [49] Q3 analysis indicated that there was no significant local dependency across the depression, anxiety, or stress scales. STR5 (item 12; “I found it difficult to relax”) showed a Yen’s [49] Q3 with STR1 (item 1; “I found it hard to wind down”) of 0.22; previous research has investigated loading the stress items on a range of different factors (eg, tripartite model and quadripartite model), so it is unsurprising to see a stress item demonstrates local dependency in this analysis, which may be due to another trait being involved; this did not warrant removal of the item given the significant literature, which supports the 21-item, 3-factor structure of the DASS-21.

### Graded Response Model

**Figure 2:**
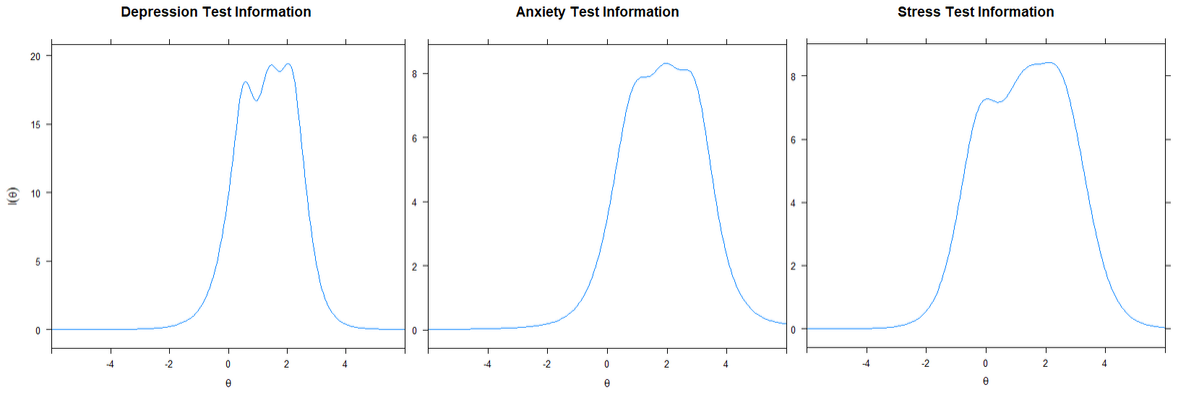
Scale test information.

All 3 scales demonstrated good fit with the graded response model across the CFI, TLI, and SRMR fit indices (Table 3). The stress scale RMSEA was worse, likely due to the unidimensional execution of this analysis, suggesting that there is residual variance unaccounted for by the “stress” latent trait. All scales demonstrated a marginal reliability above 0.7. Item parameters for interested readers are contained in Table S1 in Multimedia Appendix 1. There were no disordered thresholds across any of the items (Figures S2-S4 in Multimedia Appendix 1). The scales provide the most information at approximately 2 θ (Figure 2).

### Differential Item Functioning

Collapsed employment groups (“Employed” and “Not Employed”) and education groups (“Nontertiary” and “Tertiary”) were used for DIF. Collapsing the education groups did not result in enough nontertiary participants across response options for items anxiety 3 (item 7), anxiety 7 (item 20), and stress 6 (item 14). Otherwise, no depression, anxiety, or stress items were flagged for DIF by gender, employment, or education. There was also no differential test functioning flagged.

### CAT Simulation

At an SEM of 0.45 (approximate reliability of 0.80), the CATs required fewer items as the simulated participants’ θ increased (Table 4). As few as 1 or 2 items were required for the depression scale, and as few as 3 or 4 items for the anxiety and stress scales. Simulating a CAT with an SEM stop rule of 0.45 (Table 5) showed that across a θ of 0.0 through to 3.0 there was a reduction of approximately 43% to 62% of items administered.

**Table 4:** Computer adaptive test (CAT) simulation results.

Reliability		SEM^a^	Depression		Anxiety			Stress	
			Avg items^b^	*r* ^c^	Avg items	*r*	Avg items		*r*
**Simulated sample (n=10,000)**									
	1.00	0.00	7.000	0.906	7.000	0.845	7.000		0.913
	0.90	0.32	5.484	0.904	6.986	0.847	7.000		0.914
	0.80	0.45	3.744	0.876	5.768	0.835	4.817		0.891
	0.70	0.55	3.153	0.860	4.772	0.818	3.307		0.856
	0.60	0.63	2.925	0.856	3.917	0.787	2.289		0.826
	0.50	0.71	1.683	0.829	3.080	0.771	1.442		0.763
**Validation sample (n=248)**									
	1.00	0.00	7.000	1.000	7.000	1.000	7.000		1.000
	0.90	0.32	5.440	0.993	6.988	1.000	7.000		1.000
	0.80	0.45	3.641	0.961	5.835	0.993	4.758		0.975
	0.70	0.55	3.032	0.951	4.802	0.975	3.310		0.956
	0.60	0.63	2.810	0.949	4.036	0.934	2.250		0.938
	0.50	0.71	1.665	0.915	3.190	0.902	1.460		0.882

^a^SEM: SE of measurement.

^b^Avg items: average number of items administered.

^c^*r* is the correlation between true θ and θ as calculated by the CAT.

**Table 5:** Computer adaptive test (CAT) simulation results by participant θ with SEM^a^ of 0.45 (n=10,000).

θ group				Depression				Anxiety				Stress		
				n		Avg items^b^		n		Avg items		n		Avg items
**Simulated sample (n=10,000)**														
		Below −3.0		10		7.000		8		7.000		17		7.000
		−3 to −2.4		58		7.000		68		7.000		61		7.000
		−2.4 to −1.8		280		6.982		265		7.000		292		6.983
		−1.8 to −1.2		738		6.862		792		6.999		781		6.809
		−1.2 to −0.6		1561		6.360		1597		6.959		1647		6.113
		−0.6 to 0.0		2292		4.705		2223		6.726		2324		4.778
		0.0 to 0.6		2378		2.394		2272		5.802		2145		3.963
		0.6 to 1.2		1600		1.288		1620		4.161		1598		3.922
		1.2 to 1.8		757		1.104		818		3.331		738		3.920
		1.8 to 2.4		256		1.469		269		3.119		288		3.878
		2.4 to 3.0		57		1.895		61		3.213		95		4.158
		Above 3.0		13		2.000		7		3.429		14		4.500
**Validation sample (n=248)**														
	Below −3.0		0		N/A^c^		0		N/A		0		N/A	
	−3 to −2.4		0		N/A		0		N/A		0		N/A	
	−2.4 to −1.8		0		N/A		0		N/A		0		N/A	
	−1.8 to −1.2		0		N/A		0		N/A		37		7.000	
	−1.2 to −0.6		78		7.000		89		7.000		26		6.808	
	−.6 to 0.0		45		3.533		45		7.000		60		4.350	
	0.0 to 0.6		52		1.904		52		5.596		47		3.723	
	0.6 to 1.2		43		1.349		37		3.784		48		3.729	
	1.2 to 1.8		17		1.059		8		3.125		13		4.154	
	1.8 to 2.4		7		1.571		11		3.091		7		3.714	
	2.4 to 3.0		6		2.000		3		3.000		7		4.571	
	Above 3.0		0		N/A		3		3.333		3		5.667	

^a^SEM: standard error of measurement.

^b^Avg items: average number of items administered.

^c^N/A: not applicable.

## Discussion

This study aimed to evaluate the suitability of IRT and CAT for the DASS-21.

### Factor Structure

CFA supported the quadripartite model in both the evaluation and validation samples, consistent with Henry and Crawford [5], Osman and colleagues [6], Szabó [10], and Gomez [11]. Consistent with Page et al [27], allowing factors to share variance generally resulted in improved fit across all the tested models; however, only minimally. A model that forces the 3 factors to be orthogonal has more clinical use and is more parsimonious. This analysis did not provide any substantial evidence for removal of an item.

### Item Calibration

A bifactor IRT model did not converge (insufficient n), so focus was placed on unidimensional IRT. Overall, all assumptions of IRT (unidimensionality, monotonicity, and local independence) were met for each scale within the DASS-21.

There were some consistencies with previous research using Rasch in items of concern. DEP2 (depression scale; item 5) and DEP5 (item 16) demonstrated the poorest fit with the graded response model. Duffy and colleagues [7] found that removal of DEP5 (item 16) improved CFA fit, but this was in a 2-factor model and focused on adolescents. Additionally, Parkitny and colleagues [13] found that the same item displayed possible misfit to the Rasch model; however, given the use of a different IRT model, it is not directly comparable to the current findings. Two studies [12,14] removed DEP2 due to poor fit with the Rasch model. It seems likely that DEP2 and DEP5 relate to a lack of motivation, whereas the other depression items relate to a lack of positive affect and that the IRT models are sensitive to the way in which participants respond to these items differently.

While ANX4 (item 9) displayed a low factor loading, it demonstrated suitable monotonicity and local independence. ANX1 (item 2) and ANX5 (item 15) demonstrated a lower, but acceptable, Loevinger *H* value. These items have been flagged in other studies. Duffy and colleagues [7] correlated the error terms between ANX5 (item 15) and ANX7 (item 20) due to poor fit. Shea and colleagues [14] found that ANX1 (item 2), ANX5 (item 15), and ANX7 (item 20) displayed poor fit with the Rasch model and removed ANX1, resulting in a 6-item anxiety scale that had improved fit. Parkitny and colleagues [13] observed disordered thresholds in ANX5 (item 15) but retained the item and its original scoring structure as alterations failed to meet the Rasch model requirements. Like the depression items, it seems that these items sometimes demonstrate misfit due to their content and the samples gathered.

Within the bifactor CFA, the factor loadings for the stress items on the individual stress latent construct were generally low, largely being explained by the general negative affect latent trait. This may explain the higher RMSEA when conducting a unidimensional CFA with just the stress items—there appears to be residual variance unexplained by the stress latent trait alone. While the items measure the construct of stress, it may be that people’s stress manifests in various ways, and thus, people’s pattern of responses to this item are unpredictable. Crawford and Henry [4] noted some literature had found STR1 (item 1) to have minor misspecification, but that no previous studies demonstrated a pattern of specific item removals. Overall, the stress scale appears to be the weakest of the 3 within the DASS-21 but not sufficiently problematic to justify removal of the latent trait (such as in the tripartite model).

### CAT Simulation

All 3 scales presented as “peaked tests” and thus do not measure efficiently at the very low end of the trait spectrum. This results in the same 1 or 2 items being administered for those with high levels of depression, anxiety, or stress. Stronger correlations were found between CAT simulated θ scores and true θ scores as the participants possessed higher levels of depression, anxiety, and stress; however, this began to reverse as participants approached a θ of 3.0. This is consistent with research by Page et al [27] who found the depression scale has a ceiling effect and does not measure severe depressive symptoms such as suicidal ideation or vegetative symptoms well. For the depression scale, depending on the SEM stop rule set, fewer efficiencies are gained outside the range of approximately 0θ to 2.5θ; for anxiety and stress, this same pattern is evident outside of approximately 0.0θ to 3.0θ. The stability of these findings is compromised by only having few real participants in the upper extremes of the θ range, likely due to using a nonclinical sample.

At a reliability of 0.80, which is suitable for group-level data collection and for research purposes, simulation of a DASS-21 CAT resulted in an average 48%, 17%, and 32% reduction in items administered for each scale, respectively. Given the small number of items, using a low SEM stop rule may not always be the most appropriate. Babcock and Weiss [22] suggested that SEM stop rules are ideal for large item banks, whereas in cases with small item banks with peaked information, using a combination stop rule of SEM and minimum information is most appropriate. This allows the CAT to continue administration of items where there are suitable items available and stop administration in areas of low information. This would be particularly useful in the case of the DASS-21 as there is little need to continue administration of items for people below 0θ but some use in measuring the difference between those presenting with mild to severe symptoms. It is important, in the context of stepped care models of mental health (such as that in Australia) [68], to consider the advantage of extremely short linear scales that only use the 1 or 2 items that have the highest information versus the advantage use of CATs (which come with complexity), which can measure at a highly reliable level across a spectrum of symptoms. Although not used in this study, for practical implementation, CAT software does allow for exposure rules, which ensure that certain content areas are covered. Future studies should consider different stop rules with a DASS-21 CAT and other short CATs.

### Limitations and Future Research

All items were retained despite previous studies having suggested some item removal; this suggests that there are either sample differences and thus results across these 2 studies cannot be generalized, or that the different IRT model used results in different item fit. The sample in this study was mostly female and above the age of 50 years; thus, results are not generalizable. Additionally, some research suggests that applying an inappropriate IRT model can bias goodness of fit indices and that not all goodness of fit indices are made equal [69]. Future research should consider comparing outcomes of different IRT models in determination of item removal from the DASS-21 for CAT development.

Despite identifying that a bifactor model (the “quadripartite” model when discussing the DASS-21) was most suitable, this study used each of the scales as unidimensional measures throughout the IRT analyses, resulting in multiple unidimensional CATs. Future research with larger samples should consider the use of multidimensional IRT and CAT methodologies, which may allow further efficiencies in testing to be gained [70].

Due to the application of CAT to a set of 3 scales in a unidimensional manner, there were limited items for the algorithm to choose from. For those with severe symptomology, this results in the same 1 to 2 items being administered across each scale. By addressing sample size issues (as noted earlier), it would be increasingly possible to test a multidimensional IRT model and ensure that the true advantages of CATs are being achieved over simple implementation of extremely short scales that use only items with the highest information.

Lastly, the data used in this study were gathered from a nonclinical sample who generally displayed few symptoms of depression, anxiety, and stress. As the participants displayed levels outside the range of θ that the DASS-21 CAT measured most efficiently, there are fewer efficiencies in testing being gained. Only 25-30 participants in the validation sample possessed a trait level above 1.2θ. Future research could investigate a DASS-21 CAT with a clinical population. Further research could also be conducted into items across the θ range. Additionally, although no items were found to have DIF by gender, education, or employment, this was based on collapsed groups; replication with larger samples is required.

### Conclusions

This study shows that the DASS-21 has good internal consistency (as do each of its scales) and can be fit to the graded response model. No disordered thresholds discovered across both the evaluation and validation samples. This suggests that the response format of the DASS-21 is appropriate (ie, the 4-point scale works as intended). The outcomes of the bifactor CFA challenge previous attempts to further refine item sets for the DASS-21. Rather than removing items, simulations suggest that a DASS-21 CAT results in an average 17% to 48% reduction in items administered at a reliability of 0.8, even with a poorly targeted nonclinical sample. This suggests that a DASS-21 CAT can efficiently and accurately measure symptoms at a mild to moderate level of each trait. A DASS-21 CAT could be used in clinical practice or research alongside other CATs. CAT implementation was best for the depression scale, reflecting the stronger results of this scale in the CFA and graded response model analyses. While it may be that a DASS-21 CAT would be best used based on clinical decision-making where there is a suspicion of optimal trait levels for the CAT, the benefit of CAT is that efficiencies can be achieved on a case-by-case basis automatically by the computer rather than clinicians having to decide whether a patient undergoes a CTT or CAT version of the test. The computer will automatically decide whether the client requires every item or not. This research also demonstrates that a DASS-21 CAT would be efficient and reliable for group-level measurement of psychological distress. From a research perspective, CATs will become more common, and this study demonstrates that there is little need to develop new items for use in adaptive testing, when existing tools with strong psychometrics exist can be used as part of larger CAT batteries.

## Appendix

Multimedia Appendix 1 Supporting data for item response theory analyses and outcomes.

## Abbreviations

BDI: Beck Depression Inventory
CAT: computer adaptive test
CES-D scale: Center for Epidemiological Studies Depression scale
CFA: confirmatory factor analysis
CFI: comparative fit index
CTT: classical test theory
DASS: Depression Anxiety Stress Scale
DIF: differential item functioning
GAD-7: General Anxiety Disorder-7
IRT: item response theory
PHQ-9: Patient Health Questionnaire-9
PSQ: Perceived Stress Questionnaire
RMSEA: root-mean-square error of approximation
SEM: SE of measurement
SRMR: standardized root-mean-square residual
TLI: Tucker Lewis index

## References

[1] Lovibond SH, Lovibond PF (1995). Manual for the Depression Anxiety Stress Scales. 2nd ed.

[2] Antony MM, Bieling PJ, Cox BJ, Enns MW, Swinson RP (1998). Psychometric properties of the 42-item and 21-item versions of the Depression Anxiety Stress Scales in clinical groups and a community sample. Psychol Assess.

[3] Brown TA, Chorpita BF, Korotitsch W, Barlow DH (1997). Psychometric properties of the Depression Anxiety Stress Scales (DASS) in clinical samples. Behav Res Ther.

[4] Crawford J, Henry Julie D (2003). The Depression Anxiety Stress Scales (DASS): normative data and latent structure in a large non-clinical sample. Br J Clin Psychol.

[5] Henry JD, Crawford JR (2005). The short-form version of the Depression Anxiety Stress Scales (DASS-21): construct validity and normative data in a large non-clinical sample. Br J Clin Psychol.

[6] Osman A, Wong JL, Bagge CL, Freedenthal S, Gutierrez PM, Lozano G (2012). The Depression Anxiety Stress Scales-21 (DASS-21): further examination of dimensions, scale reliability, and correlates. J Clin Psychol.

[7] Duffy CJ, Cunningham EG, Moore SM (2005). Brief report: the factor structure of mood states in an early adolescent sample. J Adolesc.

[8] Naumova K (2022). Dimensionality and reliability of the Depression Anxiety Stress Scales 21 among adolescents in North Macedonia. Front Psychol.

[9] Reise SP, Moore TM, Haviland MG (2010). Bifactor models and rotations: exploring the extent to which multidimensional data yield univocal scale scores. J Pers Assess.

[10] Szabó M (2010). The short version of the Depression Anxiety Stress Scales (DASS-21): factor structure in a young adolescent sample. J Adolesc.

[11] Gomez R (2013). Depression Anxiety Stress Scales: factor structure and differential item functioning across women and men. Pers Individ Differ.

[12] Medvedev ON, Krägeloh CU, Titkova EA, Siegert RJ (2020). Rasch analysis and ordinal-to-interval conversion tables for the Depression, Anxiety and Stress Scale. J Health Psychol.

[13] Parkitny L, McAuley JH, Walton D, Pena Costa LO, Refshauge KM, Wand BM, Di Pietro F, Moseley GL (2012). Rasch analysis supports the use of the Depression, Anxiety, and Stress Scales to measure mood in groups but not in individuals with chronic low back pain. J Clin Epidemiol.

[14] Shea TL, Tennant A, Pallant JF (2009). Rasch model analysis of the Depression, Anxiety and Stress Scales (DASS). BMC Psychiatry.

[15] Weiss DJ (2011). Better data from better measurements using computerized adaptive testing. J Methods Meas Soc Sci.

[16] Gibbons CJ, Bower P, Lovell K, Valderas J, Skevington S (2016). Electronic quality of life assessment using computer-adaptive testing. J Med Internet Res.

[17] Baker FB, Kim SH (2017). The Basics of Item Response Theory Using R.

[18] Bonifay W (2020). Multidimensional Item Response Theory.

[19] Wardenaar KJ, Wanders RBK, Jeronimus BF, de Jonge P (2018). The psychometric properties of an internet-administered version of the Depression Anxiety and Stress Scales (DASS) in a sample of Dutch adults. J Psychopathol Behav Assess.

[20] Han KT (2016). Maximum likelihood score estimation method with fences for short-length tests and computerized adaptive tests. Appl Psychol Meas.

[21] Lu P, Zhou D, Qin S, Cong X, Zhong S (2012). The study of item selection method in CAT. Computational Intelligence and Intelligent Systems.

[22] Babcock B, Weiss DJ (2012). Termination criteria in computerized adaptive tests: do variable-length CATs provide efficient and effective measurement?. J Comput Adaptive Testing.

[23] Loe BS, Stillwell D, Gibbons C (2017). Computerized adaptive testing provides reliable and efficient depression measurement using the CES-D scale. J Med Internet Res.

[24] Devine J, Fliege H, Kocalevent R, Mierke A, Klapp BF, Rose M (2016). Evaluation of computerized adaptive tests (CATs) for longitudinal monitoring of depression, anxiety, and stress reactions. J Affect Disord.

[25] Gardner W, Shear K, Kelleher KJ, Pajer KA, Mammen O, Buysse D, Frank E (2004). Computerized adaptive measurement of depression: a simulation study. BMC Psychiatry.

[26] (2018). DASS FAQ. The University of New South Wales.

[27] Page AC, Hooke GR, Morrison DL (2007). Psychometric properties of the Depression Anxiety Stress Scales (DASS) in depressed clinical samples. Br J Clin Psychol.

[28] Kraska J (2023). Graded Response Model Analysis and Computer Adaptive Test Simulation of the DASS-21 [R].

[29] R Core Team (2020). R: a language and environment for statistical computing (version 4.0.2). R Foundation for Statistical Computing.

[30] RStudio Team (2020). RStudio: integrated development for R (version 1.3.1073). RStudio, Inc.

[31] Cronbach LJ (1951). Coefficient alpha and the internal structure of tests. Psychometrika.

[32] Revelle W (2020). psych: procedures for psychological, psychometric, and personality research (version 2.0.7). Northwestern University.

[33] Bollen KA (2016). A new incremental fit index for general structural equation models. Sociol Methods Res.

[34] Bentler PM (1990). Comparative fit indexes in structural models. Psychol Bull.

[35] Tucker LR, Lewis C (1973). A reliability coefficient for maximum likelihood factor analysis. Psychometrika.

[36] Hu L, Bentler PM (1999). Cutoff criteria for fit indexes in covariance structure analysis: conventional criteria versus new alternatives. Struct Equ Modeling.

[37] Browne MW, Cudeck R (2016). Alternative ways of assessing model fit. Sociol Methods Res.

[38] Rigdon EE (1996). CFI versus RMSEA: a comparison of two fit indexes for structural equation modeling. Struct Equ Modeling.

[39] MacCallum RC, Browne MW, Sugawara HM (1996). Power analysis and determination of sample size for covariance structure modeling. Psychol Methods.

[40] Hooper D, Coughlan J, Mullen M (2008). Structural equation modelling: guidelines for determining model fit. Electron J Bus Res Methods.

[41] Rosseel Y (2012). lavaan: an R package for structural equation modeling. J Stat Softw.

[42] Mokken RJ (1971). A Theory and Procedure of Scale Analysis: With Applications in Political Research.

[43] Loevinger J (1948). The technic of homogeneous tests compared with some aspects of scale analysis and factor analysis. Psychol Bull.

[44] Ligtvoet R, van der Ark L, te Marvelde JM, Sijtsma K (2010). Investigating an invariant item ordering for polytomously scored items. Educ Psychol Meas.

[45] Sijtsma K, Meijer RR (2016). A method for investigating the intersection of item response functions in Mokken's nonparametric IRT model. Appl Psychol Meas.

[46] Straat JH, van der Ark LA, Sijtsma K (2014). Minimum sample size requirements for Mokken scale analysis. Educ Psychol Meas.

[47] van der Ark LA (2007). Mokken scale analysis in R. J Stat Softw.

[48] van der Ark LA (2012). New developments in Mokken scale analysis in R. J Stat Softw.

[49] Yen WM (1993). Scaling performance assessments: strategies for managing local item dependence. J Educational Measurement.

[50] Christensen KB, Makransky G, Horton M (2017). Critical values for Yen's Q3: identification of local dependence in the Rasch model using residual correlations. Appl Psychol Meas.

[51] Samejima F (1970). Erratum estimation of latent ability using a response pattern of graded scores. Psychometrika.

[52] Samejima F, van der Linden WJ, Hambleton RK (1997). Graded response model. Handbook of Modern Item Response Theory.

[53] Aybek EC, Demirtasli RN (2017). Compturized adaptive test (CAT) applications and item response theory models for polytomous items. Int J Res Educ Sci.

[54] Lane S, Stone CA, Ankenmann RD, Liu M (1995). Examination of the assumptions and properties of the graded item response model: an example using a mathematics performance assessment. Appl Meas Educ.

[55] Reise SP, Cautin RL, Lilienfeld SO (2015). Item response theory. The Encyclopedia of Clinical Psychology.

[56] Yang F, Kao ST (2014). Item response theory for measurement validity. Shanghai Arch Psychiatry.

[57] Cai L, Monroe S (2014). A new statistic for evaluating item response theory models for ordinal data. CRESST Report 839. National Center for Research on Evaluation, Standards, and Student Testing (CRESST).

[58] Orlando M, Thissen D (2016). Likelihood-based item-fit indices for dichotomous item response theory models. Appl Psychol Meas.

[59] Andrich D, Luo G (2003). Conditional pairwise estimation in the Rasch model for ordered response categories using principal components. J Appl Meas.

[60] Tennant A, Penta M, Tesio L, Grimby G, Thonnard JL, Slade A, Lawton G, Simone A, Carter J, Lundgren-Nilsson A, Tripolski M (2004). Disordered thresholds: an example from the functional independence measure. Rasch Measurement Transactions.

[61] Chalmers RP (2012). mirt: a multidimensional item response theory package for the R environment. J Stat Softw.

[62] Holland PW, Wainer H (1993). Differential Item Functioning.

[63] Thissen L, Steinberg D, Wainer H, Holland PW, Wainer H (1993). Detection of differential item functioning using the parameters of item response models. Differential Item Functioning.

[64] Choi SW, Swartz RJ (2009). Comparison of CAT item selection criteria for polytomous items. Appl Psychol Meas.

[65] Huebner AR, Wang C, Quinlan K, Seubert L (2016). Item exposure control for multidimensional computer adaptive testing under maximum likelihood and expected a posteriori estimation. Behav Res Methods.

[66] Chalmers RP (2016). Generating adaptive and non-adaptive test interfaces for multidimensional item response theory applications. J Stat Soft.

[67] Szabó M, Lovibond PF (2006). Anxiety, depression, and tension/stress in children. J Psychopathol Behav Assess.

[68] Carey T (2018). Stepped care for mental health treatment. A system in need of psychological expertise. InPsych.

[69] Stone CA, Zhang B (2003). Assessing goodness of fit of item response theory models: a comparison of traditional and alternative procedures. J Educ Meas.

[70] Makransky G, Mortensen EL, Glas CAW (2013). Improving personality facet scores with multidimensional computer adaptive testing: an illustration with the NEO PI-R. Assessment.

