# Supplementary material for: Graded Response Model Analysis and Computer Adaptive Test Simulation of the Depression Anxiety Stress Scale 21: Evaluation and Validation Study
Source: J Med Internet Res. 2023 Jun 22;25:e45334. doi: 10.2196/45334 (PMC10337454; doi:10.2196/45334)

Table S1: Item parameters for the evaluation and validation samples

|  | Evaluation | | | |
| --- | --- | --- | --- | --- |
| Item | a | d1 | d2 | d3 |
| Depression |  |  |  |  |
| DEP1 | 3.567 | -2.012 | -5.560 | -8.328 |
| DEP2 | 1.707 | 0.649 | -2.043 | -3.849 |
| DEP3 | 4.510 | -2.304 | -6.013 | -9.352 |
| DEP4 | 2.956 | 0.142 | -3.490 | -6.358 |
| DEP5 | 2.667 | -0.831 | -4.241 | -6.492 |
| DEP6 | 2.666 | -1.258 | -3.824 | -5.670 |
| DEP7 | 3.703 | -2.449 | -5.591 | -8.090 |
| Anxiety |  |  |  |  |
| ANX1 | -1.536 | -1.311 | -3.155 | -4.971 |
| ANX2 | 2.570 | -2.549 | -5.125 | -7.265 |
| ANX3 | 2.568 | -2.696 | -5.127 | -7.644 |
| ANX4 | 1.647 | -1.166 | -2.950 | -4.921 |
| ANX5 | 0.656 | 0.154 | -2.293 | -3.981 |
| ANX6 | 1.948 | -0.697 | -3.228 | -5.607 |
| ANX7 | 2.604 | -1.837 | -4.869 | -7.469 |
| Stress |  |  |  |  |
| STR1 | 1.999 | 0.615 | -2.050 | -4.500 |
| STR2 | 2.054 | 0.042 | -3.449 | -5.756 |
| STR3 | 1.954 | -0.337 | -2.943 | -5.303 |
| STR4 | 2.360 | 0.687 | -3.129 | -5.750 |
| STR5 | 2.247 | 0.640 | -2.449 | -5.261 |
| STR6 | 2.086 | -1.744 | -4.197 | -6.699 |
| STR7 | 1.954 | 0.400 | -3.101 | -5.445 |

Figure S1. Category Characteristic Curve


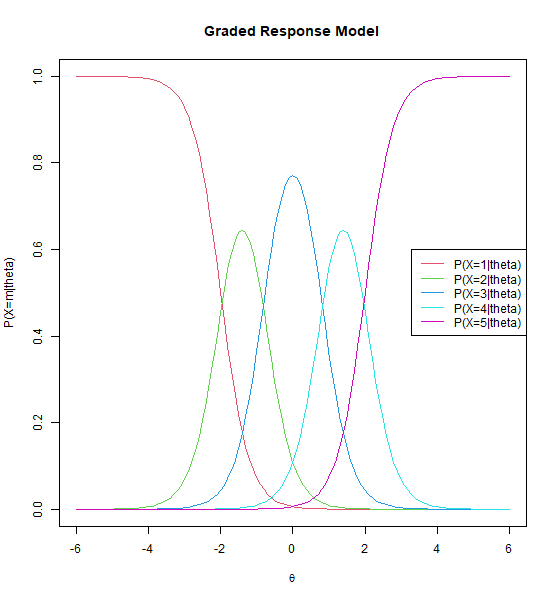


Figure S2. Depression Category Characteristic Curve


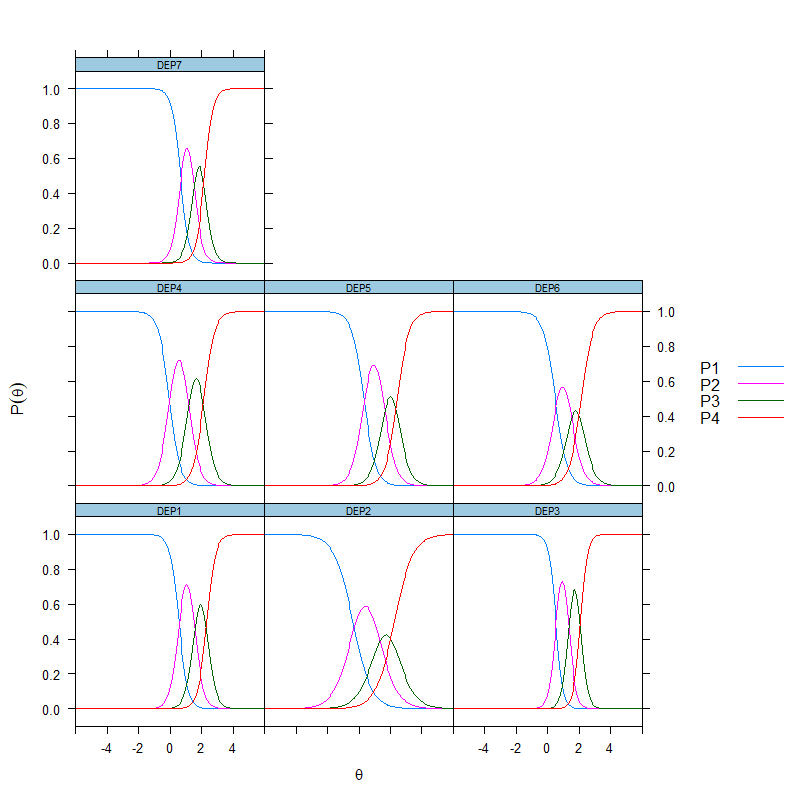


Figure S3. Anxiety Category Characteristic Curve


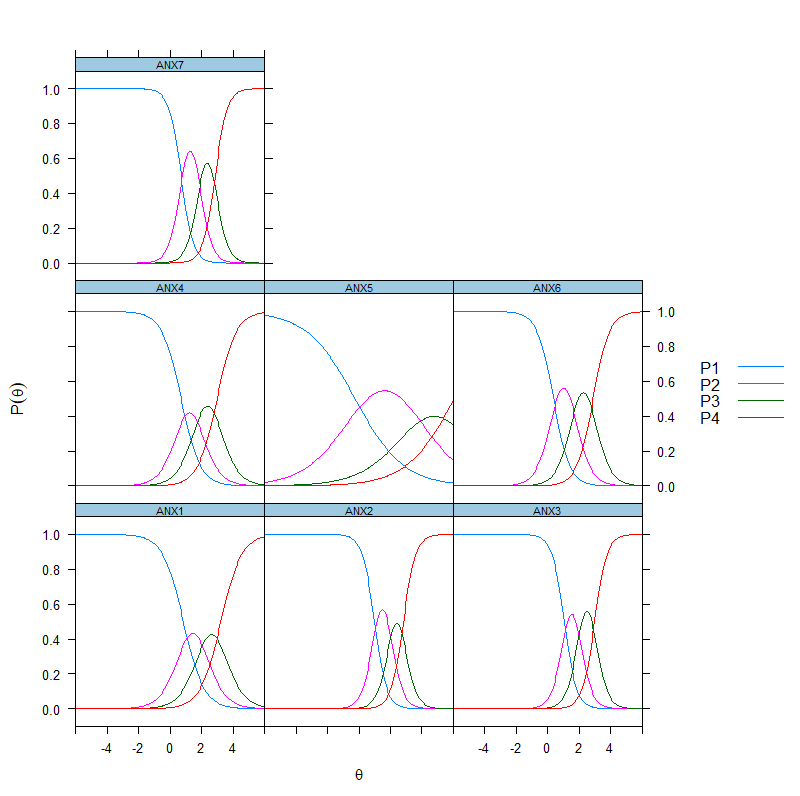


Figure S4. Stress Category Characteristic Curve


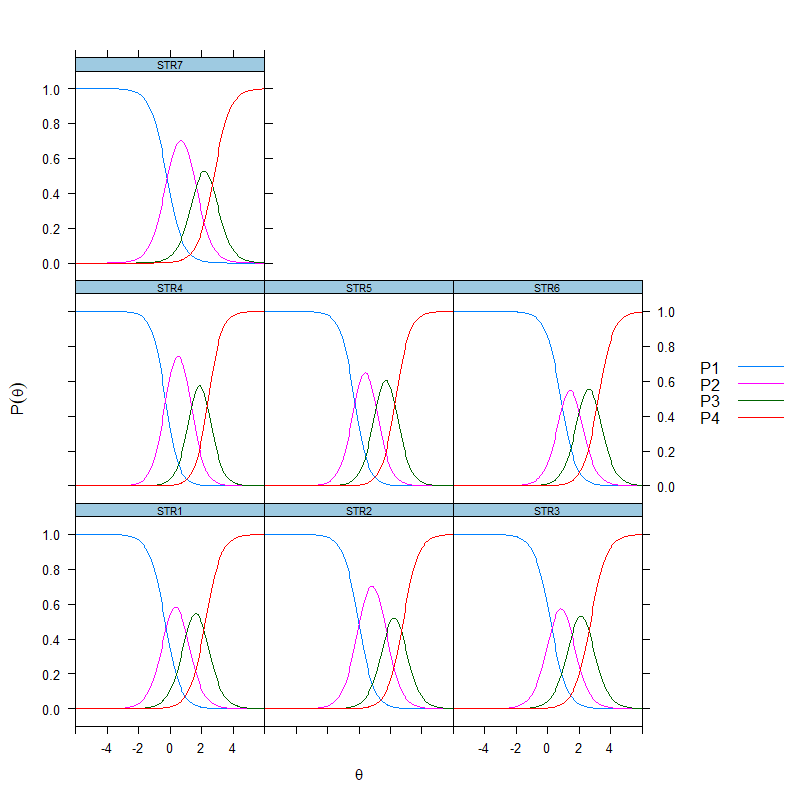

Supplement: Multimedia Appendix 1 [file jmir_v25i1e45334_app1.docx]
